# Fitz-Hugh-Curtis Syndrome: An Incidental Diagnostic Finding During Laparoscopic Sterilization

**DOI:** 10.7759/cureus.10304

**Published:** 2020-09-08

**Authors:** Georgios Grigoriadis, Jacinda Green, Allison Amin, Deborah Cross

**Affiliations:** 1 Obstetrics and Gynaecology, Hull Royal Infirmary, Hull, GBR; 2 Obstetrics and Gynaecology, Harrogate and District NHS Foundation Trust, Harrogate, GBR

**Keywords:** tuberculosis, laparoscopy, sterilization, adhesions, fitz-hugh-curtis

## Abstract

Fitz-Hugh-Curtis syndrome is associated with the presence of perihepatic adhesions, with or without associated symptoms. It is, most commonly, a result of chlamydial or gonococcal infections. We present a case of Fitz-Hugh-Curtis syndrome in an asymptomatic female patient who underwent a routine laparoscopic sterilization. The patient had, otherwise, normal intraoperative findings and negative laboratory investigations. The patient had a past history of tuberculosis infection, for which she had received treatment 10 years ago. We also present the literature relevant to our case report.

## Introduction

"Fitz-Hugh-Curtis" (FHC) syndrome, also known as acute perihepatitis, typically presents with "violin-string" adhesions between the liver and the anterior abdominal wall or the diaphragm [1], most commonly as a result of chlamydial or gonococcal infections [2]. Patients can be asymptomatic or present with clinical symptoms, such as pain and/or fever [3]. There have been reports of the syndrome in patients with genital and peritoneal tuberculosis (TB) [4-7].

We present a case of FHC syndrome in an asymptomatic female patient with past history of extrapulmonary TB. The syndrome was diagnosed incidentally during routine laparoscopic sterilization, with otherwise normal intraoperative findings and negative laboratory tests for chlamydial or gonococcal infections.

## Case presentation

A 38-year-old patient, para 2 (two previous normal deliveries), of south-Asian origin, was seen in the gynaecology outpatient department requesting sterilization. She had the contraceptive implant in situ, which had been inserted 13 months ago. However, she was not keen to continue with the same method of contraception. She was amenorrhoeic, her body mass index (BMI) was 27.3, and there was no past medical or surgical history documented by the gynaecologist who reviewed her in the outpatient department. She was not on any regular medication and no drug allergies were noted. The procedure of laparoscopic sterilization (including risks and benefits) was explained to the patient, alternative contraceptive options discussed, written consent was obtained, and the patient was booked for laparoscopic sterilization and removal of contraceptive implant under general anaesthesia. She had routine preoperative assessment where it was noted that she had previously suffered from abdominal TB for which she had received medical treatment in a large teaching hospital in 2010.

**Figure 1:**
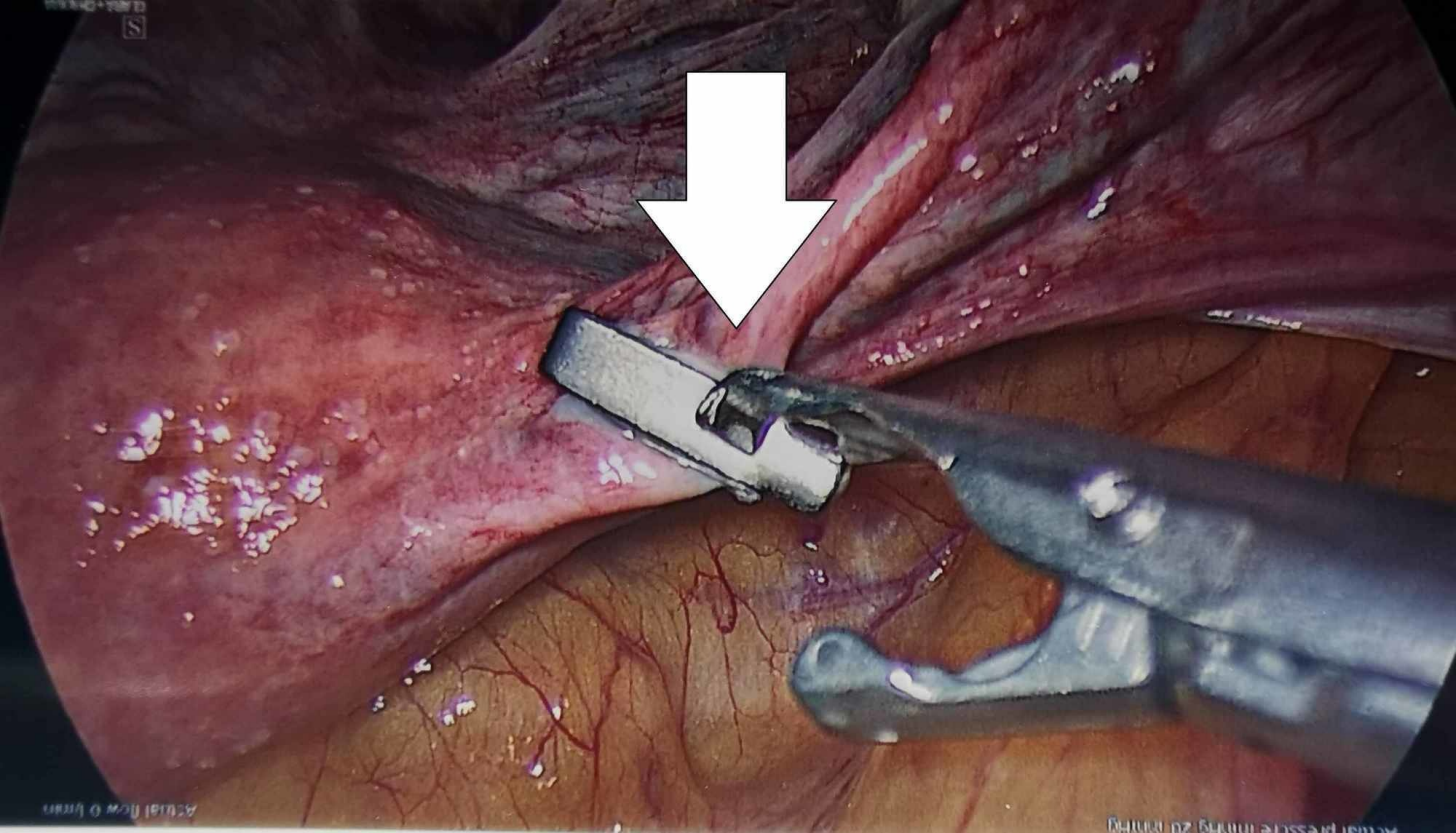
Right fallopian tube with Filshie clip applied (arrow)

The patient was admitted for the above day-case procedure two months following initial review. A routine two-port laparoscopy was performed and one Filshie clip was applied on each fallopian tube (Figure 1).

**Figure 2:**
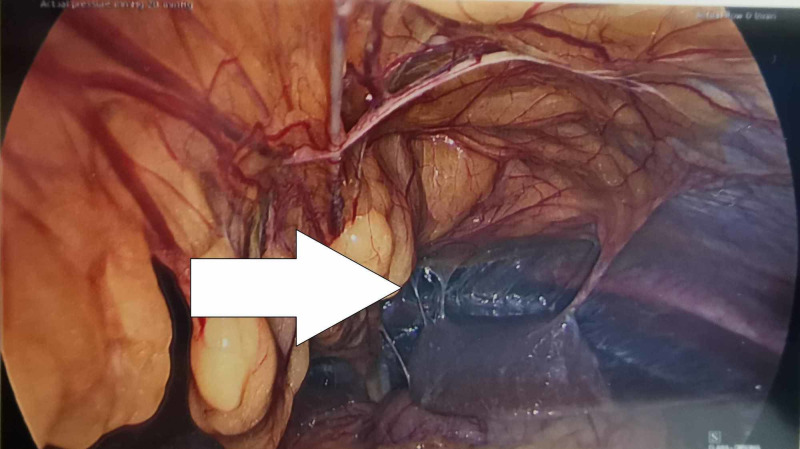
Perihepatic adhesions (arrow)

**Figure 3:**
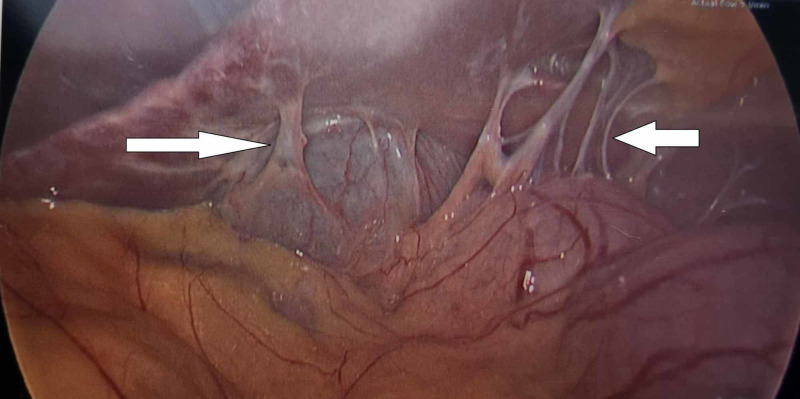
More perihepatic adhesions (arrows)

"Violin-string" perihepatic adhesions were noted, in keeping with Fitz-Hugh-Curtis syndrome (Figures 2, 3). 

The rest of the abdominal and pelvic anatomy was normal. A small amount of clear, free fluid was noted in the pouch of Douglas and aspirated. Postoperative blood tests showed normal inflammatory markers. Vaginal and endocervical swabs were performed and were negative for Chlamydia trachomatis ribosomal ribonucleic acid (rRNA) and Neisseria gonorrhoeae rRNA. No pathogens were isolated. The free peritoneal fluid was tested: microscopy for alcohol and acid-fast bacilli (AAFB) was negative, polymerase chain reaction (PCR) testing for the deoxyribonucleic acid (DNA) from Mycobacterium tuberculosis was negative, and culture for Mycobacterium tuberculosis was also negative.

## Discussion

The syndrome was first described by Carlos Stajano in 1920 [8]. Arthur Hale Curtis, in 1930, described the presence of "violin-string" adhesions between the liver and the anterior abdominal wall in patients with evidence of gonococcal salpingitis [9] and, four years later, Thomas Fitz-Hugh Jr reported three cases of "acute gonococcal peritonitis" of the right upper quadrant [10] to complete the clinical picture. Incidence of FHC syndrome in the general population is unknown. For symptomatic patients, clinical presentation can mimic conditions such as acute cholecystitis, gallbladder stones, acute appendicitis, acute pyelonephritis, acute hepatitis, acute gastritis, or gastric ulcer [11].

Although diagnosis is usually made following laparoscopy or laparotomy, enhanced CT imaging can be a useful, non-invasive diagnostic modality [12]. Presence of concomitant ascites has been described and can be picked up by ultrasound imaging [13]. In a prospective study from India over a four-year period, 320 female patients underwent diagnostic laparoscopy for sterilization, infertility or chronic pelvic pain, and the incidental finding of FHC syndrome was observed in 8%, 16.6%, and 30%, respectively [14]. 

Sharma et al. reported three cases of FHC syndrome as a result of proven genital TB, all of which had pain and/or infertility [4]. Coremans and de Clerck reported a case of FHC syndrome associated with tuberculous salpingitis and peritonitis where ascites was the primary clinical finding [5]. Jose et al. described a case of FHC syndrome as primary manifestation in an immune-competent female patient with disseminated TB [6]. In a study of 82 patients with genital TB who had diagnostic laparoscopy for infertility or chronic pelvic pain or tubo-ovarian masses, 48.7% were found to have evidence of FHC syndrome [7].

Extrapulmonary TB is a rare manifestation of TB. Within this context, the peritoneum remains one of the most common sites and tuberculous peritonitis should be suspected in all patients with unexplained lymphocytic ascites [15]. Peritoneal TB can mimic other conditions such as ovarian malignancy [16] and peritoneal carcinomatosis [17].

For the definitive diagnosis of tuberculous peritonitis, microbiological and/or histological confirmation is indicated [18]. A low threshold for diagnostic laparoscopy is recommended [15], as this allows direct visualisation of the peritoneal surfaces and ascitic fluid/peritoneal biopsies to be obtained at the same time. During laparoscopic examination, the infected peritoneal surfaces can have whitish nodules, and histological examination reveals caseating or non-caseating granulomatous inflammation in more than 95% of cases [19]. In our patient, all microbiology was negative as the TB had been treated 10 years ago and laparoscopy did not reveal any nodules.

## Conclusions

We presented a clinical case of a patient with an incidental finding of FHC syndrome associated with a previous history of extrapulmonary TB. Whilst peritoneal TB remains a rare manifestation of TB, it is the most common extrapulmonary site for this infection. This is therefore a diagnosis to consider in a patient with FHC syndrome where there is no evidence of previous chlamydial or gonoccocal infections, particularly as prevalence of abdominal tuberculous infection is increasing in both developing and developed countries.
